# Endocrine responses to diverse stressors of capture, entanglement and stranding in leatherback turtles (*Dermochelys coriacea*)

**DOI:** 10.1093/conphys/cow022

**Published:** 2016-06-22

**Authors:** Kathleen E. Hunt, Charles J. Innis, Constance Merigo, Rosalind M. Rolland

**Affiliations:** 1 John H. Prescott Marine Laboratory, Research Department, New England Aquarium, Boston, MA 02110, USA; 2 Animal Health Department, New England Aquarium, Boston, MA 02110, USA; 3 Rescue and Rehabilitation Department, New England Aquarium, Boston, MA 02110, USA

**Keywords:** Anthropogenic stressors, corticosterone, *Dermochelys coriacea*, sea turtles, stress, thyroxine

## Abstract

Leatherback turtles (*Dermochelys coriacea*) are exposed to many anthropogenic stressors, yet almost no data on stress physiology exist for this species. As a first step toward understanding the physiological responses of leatherback turtles to stress, and with the particular goal of assessment of the effect of capture, we quantified corticosterone (an adrenal stress hormone) and thyroxine (a regulator of metabolic rate, often inhibited by chronic stress) in 17 healthy leatherback turtles captured at sea for scientific study, with comparisons to 15 ‘distressed’ leatherbacks that were found entangled in fishing gear (*n* = 8), confined in a weir net (*n* = 1) or stranded on shore (*n* = 6). Distressed leatherbacks had significantly elevated corticosterone (mean ± SEM 10.05 ± 1.72 ng/ml, median 8.38 ng/ml) and free thyroxine (mean 0.86 ± 0.37 pg/ml, median 0.08 pg/ml) compared with healthy leatherbacks sampled immediately before release (after ∼40 min of handling; corticosterone, mean 4.97 ± 0.62 ng/ml, median 5.21 ng/ml; and free thyroxine, mean 0.05 ± 0.05 pg/ml, median 0.00 pg/ml). The elevated thyroxine in distressed turtles compared with healthy turtles might indicate an energetic burden of entanglement and stranding. Six of the healthy leatherbacks were sampled twice, at ∼25 and ∼50 min after the time of first disturbance. In all six individuals, corticosterone was higher in the later sample (earlier sample, mean 2.74 ± 0.88 ng/ml, median 2.61 ng/ml; later sample, mean 5.43 ± 1.29 ng/ml, median 5.38 ng/ml), indicating that capture and handling elicit an adrenal stress response in this species. However, the corticosterone elevation after capture appeared relatively mild compared with the corticosterone concentrations of the entangled and stranded turtles. The findings suggest that capture and handling using the protocols described (e.g. capture duration <1 h) might represent only a mild stressor, whereas entanglement and stranding might represent moderate to severe stressors.

## Abstract (precis)

Limited data on stress physiology exist for leatherback turtles. Examination of 32 leatherback turtles showed that individuals exposed to entanglement, stranding, or entrapment in a weir net had elevated corticosterone and thyroxine compared to healthy controls.

## Introduction

Leatherback turtles (*Dermochelys coriacea*), the largest species of turtle, are considered ‘vulnerable’ by the World Conservation Union (Wallace *et al.*, 2013) and are listed as ‘endangered’ in the USA (National Oceanographic and Atmospheric Administration, 2014). They are subjected to a wide variety of potential anthropogenic stressors, including entanglement in fishing gear, boat strikes, anthropogenic disturbance and intentional capture for scientific research (e.g. Godley *et al.*, 1998; Lewison *et al.*, 2004; James *et al.*, 2005; Alfaro-Shigueto *et al.*, 2007). For management purposes, it would be useful to understand the relative impacts of these different stressors on the physiology of leatherback turtles, but because of the great size of this species (adults often weigh several hundred kilograms) and difficulty of capturing them, only limited physiological data have been published for leatherback turtles at sea (Innis *et al.*, 2010, 2014; Harris *et al.*, 2011). For example, studies using intentional capture (e.g. to attach tags for satellite telemetry) have provided substantial insight into the natural history, ecology and health of the species (James and Mrosovsky, 2004; James *et al.*, 2005, 2007; Doyle *et al.*, 2008; Innis *et al.*, 2010; Benson *et al.*, 2011; Dodge *et al.*, 2011, 2014, 2015; Harris *et al.*, 2011), but potential impacts of capture on stress physiology have rarely been assessed (Innis *et al.*, 2014).

The adrenal hormone corticosterone is widely used in wildlife stress research as an index of the overall physiological impact of anthropogenic and natural stressors (Sheriff *et al.*, 2011; Dantzer *et al.*, 2014; Madliger and Love, 2015; Madliger *et al.*, 2016). In most vertebrates, exposure to any of a variety of stressors (injury, disease, starvation, capture, etc.) triggers an endocrine ‘stress response’ characterized by rapid activation of the hypothalamic–pituitary–adrenal axis, resulting in elevation of corticosterone (and/or cortisol), along with downstream alterations in other hormones, such as thyroid hormones (Wingfield *et al.*, 1997; Wingfield and Romero, 2011; Sapolsky *et al.*, 2000; Romero and Wingfield, 2016). The glucocorticoids orchestrate a systemic response to unpredictable events, redirecting available energy and behaviour toward escape, foraging and other coping strategies and inhibiting less urgent processes, such as growth and reproduction (Wingfield *et al.*, 1997; Wingfield and Romero, 2011; Sapolsky *et al.*, 2000; Romero and Wingfield, 2016). Available data indicate that sea turtles have a robust stress response, with corticosterone elevating sharply in response to stressors such as entanglement in fishing gear, removal from water, ‘turning stress’ (turtle turned upside-down), capture and handling, fibropapillomatosis, laparoscopy, cold-stunning, osmotic stress, heat stress, injury and transportation (Gregory *et al.*, 1996; Valverde *et al.*, 1999; Hoopes *et al.*, 2000; Ortiz *et al.*, 2000; Jessop *et al.*, 2000, 2004b, c; Gregory and Schmid, 2001; Jessop and Hamann, 2005; Blanvillain *et al.*, 2008; Snoddy *et al.*, 2009, Hunt *et al.*, 2016). For leatherback turtles, however, adrenal physiology remains almost entirely unstudied. Baseline corticosterone in this species has not been well characterized, and the effect of exposure to anthropogenic disturbance, including capture for scientific studies, is unknown. Corticosterone concentrations have been reported only from two studies of nesting females (Rostal *et al.*, 2001; Deem *et al.*, 2006), and no endocrine information is available from adult leatherbacks at sea.

Thyroid hormones also respond to stress and are increasingly being studied as a complement to corticosterone (e.g. Ayres *et al.*, 2012; Joly *et al.*, 2015). The thyroid gland releases the pro-hormone thyroxine (T4), which is converted by target tissues to the active hormone tri-iodothyronine (T3). Both T3 and T4 can circulate bound to carrier proteins in plasma or can circulate ‘free’ (unbound), with only the free portion having biological effects (Norris, 2006). Free T3 concentrations are typically very low in vertebrates and are often undetectable (Kohel *et al.*, 2001; Norris, 2006), but free T4 (‘fT4’) is usually detectable in sea turtle plasma (e.g. Hunt *et al.*, 2012). Generally, the thyroid hormones affect metabolic rate, typically increasing during periods of heightened activity and increased metabolic demand (e.g. migration, moult and exercise; John-Alder, 1990; Hulbert, 2000; Mastorakos and Pavlatou, 2005) and decreasing when energy must be conserved, such as during chronic stress and, in particular, nutritional stress (Eales, 1988; Bentley, 1998; Moon *et al.*, 1999). Although thyroid hormones in ectotherms have been less studied, positive correlations of thyroid hormones with activity have been documented in several turtle species (Licht *et al.*, 1985; Southwood and Avens, 2010), including at least one sea turtle, the Kemp’s ridley (Hunt *et al.*, 2012), as well as in other reptile taxa (Gerwien and John-Alder, 1992; Rivera and Lock, 2008). Leatherbacks, additionally, are endothermic; it is common for their body temperature to exceed that of their ambient environment by as much as 18°C, although differentials of 6–8°C are more typical (Frair *et al.*, 1972; James and Mrosovsky, 2004; Innis *et al.*, 2010). Leatherback turtles thus might be expected to have direct relationships between thyroid hormones and metabolic rate, as is the case in other endotherms. However, only one study (on nesting females) has evaluated thyroid hormones in leatherback turtles (Perrault *et al.*, 2012). As with corticosterone, no data on thyroxine are available from leatherbacks at sea, and potential effects of various stressors have not been assessed.

As a first step toward understanding the stress physiology of adult, non-nesting leatherbacks, we used archived leatherback plasma samples to compare corticosterone (as a non-specific measure of general stress) and fT4 (as a potential index of metabolic rate and/or activity) in healthy, intentionally captured leatherbacks (hereafter termed ‘healthy’ leatherbacks) vs. leatherbacks that were entangled in fishing gear, stranded on shore or entrapped in a weir net (hereafter termed ‘distressed’ leatherbacks). A primary goal of this study was to establish whether capture of live leatherbacks at sea is a serious stressor, assessed as follows: (i) by comparison of corticosterone of healthy turtles at the end of the handling event with corticosterone of distressed turtles; and (ii) by assessment of the rate and degree of increase in corticosterone from a first sample taken immediately post-capture to a second sample taken immediately before release, in order to determine whether leatherbacks mount an adrenal response as a result of capture. A secondary goal was to test whether fT4 shows any correlation with known stressors.

## Materials and methods

### Healthy leatherbacks: capture and sampling

Seventeen leatherback turtles were captured off the coast of Massachusetts in 2007, 2008, 2009 and 2012 as part of an ecology and health assessment study, details of which have been described previously (Innis *et al.*, 2010, 2014; Dodge *et al.*, 2011, 2014, 2015). The location, sex, date of sampling, body temperature, ambient water temperature (where available), curved carapace length and venipuncture site are presented for all turtles in Table 1. Briefly, boat and aerial surveys were used to locate turtles resting at the sea surface, and a break-away hoop net with a purse-string closure was used to capture the turtle from the bow of a vessel. The turtle was then secured on a ramp deployed from the stern and brought onto the deck. Blood sampling was timed from the moment the hoop net was deployed (‘time zero’). Turtles did not appear to react behaviourally to vessel approach until the net was deployed, and therefore we assumed that turtles experienced minimal to no stress before net deployment. Once secured on deck, turtles were physically examined, a satellite tag was attached to the carapace, and blood samples were collected. Venipuncture sites were disinfected using sterile povidone iodine and isopropyl alcohol-infused gauze pads, and a 3–40 ml blood sample was collected from the jugular vein or dorsal tail vein using a 1.5–3 inch (3.8–7.6 cm), 18–21 gauge needle attached to a heparinized syringe (Table 1). Syringes were prepared using liquid sodium heparin (heparin sodium, 1000 USP/ml; APP Pharmaceuticals, LLC, Schaumburg, IL, USA), which was repeatedly expelled from the syringe until no visible heparin remained, resulting in a heparin concentration of <10 USP per millilitre of blood (Innis *et al.*, 2010). Whole blood was then transferred to lithium heparin blood collection tubes (BD Vacutainer; Beckton Dickinson and Co., Franklin Lakes, NJ, USA).

**Table 1:** Identification number, date, status, location (US state), sex, length, mass, body temperature, water temperature and venipuncture site for 32 leatherback turtles (*Dermochelys coriacea*) for which plasma hormone data were evaluated

Turtle no.	Date (day/month/year)	Status	US state	Sex	CCL (cm)	Mass (kg)	TB (°C)	TW (°C)	Blood site
04-07	19/08/2007	E	MA	M	141	NR	NR	21.6	J
05-07	29/08/2007	E	MA	M	143	NR	25.4	22.8	NR
06-07	29/08/2007	E	MA	U	123	NR	26.2	21.4	NR
07-07	22/09/2007	E	MA	U	138	NR	25.0	18.0	NR
08-07	01/10/2007	E	MA	F	136	NR	23.3	17.7	T
01-08	17/07/2008	C	MA	M	150	NR	30.0	25.1	NR
02-08	26/07/2008	C	MA	F	146	NR	28.8	19.5	NR
03-08	29/07/2008	C	MA	F	162	NR	29.5	20.5	NR
04-08	10/08/2008	C	MA	M	152	NR	26.7	18.8	NR
05-08	10/08/2008	C	MA	U	140	NR	28.9	19.0	NR
06-08	10/08/2008	C	MA	U	134	NR	26.7	19.0	J
07-08	10/08/2008	C	MA	M	153	NR	27.2	18.8	T
08-08	21/08/2008	C	MA	F	145	NR	24.5	19.8	NR
10-08	22/08/2008	C	MA	M	139	NR	26.3	20.3	T
11-08	23/08/2008	E	MA	M	146	NR	26.3	23.0	J
12-08	08/28/2008	E	MA	U	140	NR	26.6	21.7	J
02-09	10/07/2009	W	MA	U	127	NR	23.6	20.3	NR
04-09	27/08/2009	C	MA	U	128	NR	29.0	NR	NR
05-09	09/03/2009	E	MA	U	155	NR	29.0	NR	T
NEST11007	05/11/2011	S	MA	F	137	180	10.7	NR	T
01-12	02/08/2012	C	MA	U	137	NR	29.4	23.2	T
02-12	02/08/2012	C	MA	F	148	NR	30.1	24.2	T
03-12	08/08/2012	C	MA	F	156	NR	27.3	20.3	T
04-12	08/08/2012	C	MA	M	152	NR	27.3	20.7	T
05-12	09/08/2012	C	MA	F	156	NR	28.2	20.2	J
06-12	09/08/2012	C	MA	M	144	NR	25.8	20.3	J
07-12	09/08/2012	C	MA	F	153	NR	27.9	20.5	J
NEST12016	20/09/2012	S	MA	M	150	297	20.1	NR	J
NEST13002	11/09/2013	S	MA	F	163	NR	22.7	23.8	J
140131-01	31/01/2014	S	NC	F	154	319	NR	NR	J
140305-01	05/03/2014	S	NC	F	161	358	NR	NR	J
140308-02	08/03/2014	S	NC	F	155	310	12.6	9.0	J

Abbreviations: C, captured; CCL, curved carapace length; E, entangled; F, female; J, jugular vein; M, male; MA, Massachusetts; NC, North Carolina; NR, not recorded; S, stranded; T, dorsal tail vein; TB, body temperature; TW, water temperature; U, unknown sex; W, weir net.

In most years, restrictions on research permits allowed collection of only one blood sample from each turtle. This sample was typically collected after completion of physical examination and satellite tagging, i.e. immediately before release, and is termed the ‘Release’ sample. Release samples were collected on average 39.3 ± 10.4 min (mean ± SD) after time zero. In 2012, permission was obtained to collect an earlier blood sample as well, for comparison with Release samples, in order to assess whether physiological changes occurred during handling (Innis *et al*., 2014). These earlier samples, termed ‘Capture’ samples, were collected from six turtles as quickly as possible after time zero. In the 2012 turtles, Capture samples were collected 25.9 ± 7.2 min (mean ± SD) after time zero and Release samples 50.57 ± 9.29 min (mean ± SD) after time zero.

Six turtles were classified as male and seven as female based on tail morphology or visibly extruded penis, whereas four could not be sexed because of relatively smaller size (based on James *et al.*, 2007; Table 1). Data from males, females and animals of unknown sex were combined after testing to verify that there were no significant sex differences in either hormone; none were found [ANOVAs on male vs. female vs. unknown sex, and *t*-tests on male vs. female (excluding unknown sex); both tests were repeated for healthy turtles only, distressed turtles only and all turtles, and for both hormones; all *P*-values >0.05, details not shown].

All 17 of the intentionally captured leatherbacks were judged by the attending veterinarian to be in good health. Details of the general health status, physiological status, and post-release monitoring of most of these turtles have been reported previously (Innis *et al.*, 2010, 2014; Dodge *et al.*, 2014, 2015) and support the categorization of the intentionally captured turtles as ‘healthy’.

### Distressed leatherbacks: stranded, entangled or entrapped in weir net

Fifteen ‘distressed’ leatherbacks were evaluated between 2007 and 2013, either as part of the study described above or as part of the routine stranding response in Massachusetts and North Carolina. Turtles were considered distressed if there was clear exposure to a potential stressor known to have health consequences (from studies in other sea turtles; see above), including being entangled in fishing gear (*n* = 8), stranded on shore (*n* = 6) or entrapped in a weir net (i.e. confined in a small space but not entangled; *n* = 1).

Entangled and entrapped turtles were freed from the fishing gear, evaluated and released (for details, see Innis *et al.*, 2010; Dodge *et al.*, 2014, 2015). Stranded turtles were either hospitalized for attempted rehabilitation (*n* = 3) or humanely euthanized if found to be moribund (*n* = 3). Two of the hospitalized turtles subsequently died, whereas one individual was released (Innis *et al*., 2013).

### Blood sample processing

Blood samples were handled and processed as previously described (Innis *et al.*, 2010). Briefly, blood samples collected on board boats (healthy turtles and entangled turtles) were placed on ice packs and typically centrifuged promptly while still on the boat (median = 35 min from collection, 1500***g*** for 5 min at room temperature), with plasma then transferred to cryovials, stored on ice packs for the remainder of the day, and transferred to −20°C storage upon return to shore that evening. Samples collected on shore (stranded turtles) were typically centrifuged within 4 h (800***g*** for 20 min at 4°C for North Carolina strandings; 1500***g*** for 5 min at room temperature for Massachusetts strandings), with plasma transferred to −20 or −80°C storage immediately. All plasma samples were then archived at −80°C until analysis; sample shipments, when necessary, used dry ice. Samples from 2007–2009 were analysed at the University of Portland, OR, USA; samples from 2010–2013 were analysed at the New England Aquarium in Boston, MA, USA. All hormone assays were performed by the same scientist (K.E.H.).

### Hormone assays

Unextracted plasma samples were assayed for corticosterone with a double-antibody ^125^I radioimmunoassay (RIA) kit, and for fT4 with a coated-tube ^125^I RIA kit (both from MP Biomedicals, Solon, OH, USA; corticosterone, catalogue no. 07-120103; fT4, catalogue no. 06B-257214). These kits were selected based on successful use in other species of sea turtles (Hunt *et al.*, 2012, 2016). The manufacturer’s protocols were used except that the corticosterone assay was run at half-volume, and an additional low-dose standard was added to both hormone assays. All samples and standards were assayed in duplicate and results averaged. Any samples with coefficient of variation >10% were re-analysed to confirm results. Inter- and intra-assay precisions for both kits were below 10%; for further details, including antibody cross-reactivities, see Hunt *et al.* (2012).

Both assays were validated for unextracted leatherback plasma using standard parallelism and accuracy tests (Grotjan and Keel, 1996; Ezan and Grassi, 2000; Hunt *et al.*, 2012). Unextracted plasma was tested (rather than steroids extracted from plasma) for comparability with previous leatherback studies that also have used unextracted plasma (e.g. Rostal *et al.* 2001, Deem *et al.*, 2006) and also to avoid the additional variation that is introduced by an extraction step (see, for example, Hunt *et al.*, 2004). Note also that recent reports indicate that ether extraction of sea turtle plasma may not remove the majority of hormone from binding proteins and does not improve or alter relative patterns in data (Graham *et al.*, 2016).

For the parallelism test, serial dilutions of a leatherback plasma pool (produced by combining equal volumes from all 2007–2009 samples) were assayed alongside a full standard curve and assessed for parallelism of the linear portion of the curves. Based on parallelism results, all samples were diluted 1:10 in assay buffer for subsequent corticosterone assays, but were assayed at 1:1 for fT4, so as to fall close to 50% bound on the standard curve of each assay, the area of greatest precision. Assay accuracy (also known as ‘matrix test’) was tested by assaying standards that were spiked with an equal volume of a charcoal-stripped 1:10 pool (corticosterone) or 1:1 pool (fT4) and assessing a graph of apparent vs. expected dose for a straight slope that is close to 1.0.

### Statistical analysis

Data were not normally distributed; corticosterone data were normalized with logarithmic transformation (log[ng/ml + 1]) and analysed with parametric tests. However, fT4 data could not be normalized with common transformations (because many samples had undetectable fT4) and were therefore analysed with non-parametric tests. Samples with undetectable fT4 were assigned zeros for statistical analysis. Fisher’s exact test was also used to compare the proportion of healthy vs. distressed leatherbacks that had detectable fT4. For those healthy turtles that had two samples taken, corticosterone concentrations of Capture and Release samples were compared with Student’s paired *t*-test. Relationships of corticosterone with handling time were explored with linear regressions (independent variable was minutes since time zero; dependent variable was corticosterone) in three analyses, the first combining all available samples from healthy turtles, the second on only those samples from turtles that were sampled once, and the third on samples from turtles that were sampled twice. Comparisons of healthy with distressed turtles were performed with Student’s unpaired *t*-tests (corticosterone) or Mann–Whitney *U*-tests (fT4); for turtles that had two samples taken, only the Release sample was used in this analysis. Within the distressed group, entangled and stranded turtles were further compared with Student’s unpaired *t*-tests or Mann–Whitney *U*-tests; data from the single entrapped turtle are also presented for comparison but were not analysed statistically. One-tailed tests were used to test relationships of corticosterone with handling time because of the existence of a strong *a priori* prediction for this question; all other comparisons did not have *a priori* predictions and hence were analysed with two-tailed tests. We report means (±SEM) as well as medians in the text in order to enable comparisons with existing literature, but owing to the heteroscedasticity of most of the data, figures show medians and interquartile ranges. Assay validation results were assessed with *F*-tests for parallelism and linear regression for accuracy. All statistical testing was performed with Prism 6 for Macintosh OSX (GraphPad Software Inc., San Diego, CA, USA).

## Results

### Assay validations

Parallelism and accuracy were good for both assays. In the parallelism tests, the slope of serially diluted plasma was parallel to the standard curve (corticosterone, *F*_1,8_ = 0.1689, *P* = 0.6919; and fT4, *F*_1,5_ = 3.9425, *P* = 0.1038). In the accuracy tests, the slope of observed vs. expected dose was linear and within the desired range of 0.7–1.3 (corticosterone, *r*^2^ = 0.9677, slope = 0.8428; and fT4, *r*^2^ = 1.000, slope = 1.262).

### Healthy vs. distressed turtles

Corticosterone was detectable in all but one sample. However, fT4 was much less likely to be detectable in healthy turtles than in distressed turtles, and this difference was significant (eight of 15 distressed turtles and one of 17 healthy turtles had detectable fT4; *P* = 0.0059).

**Figure 1:**
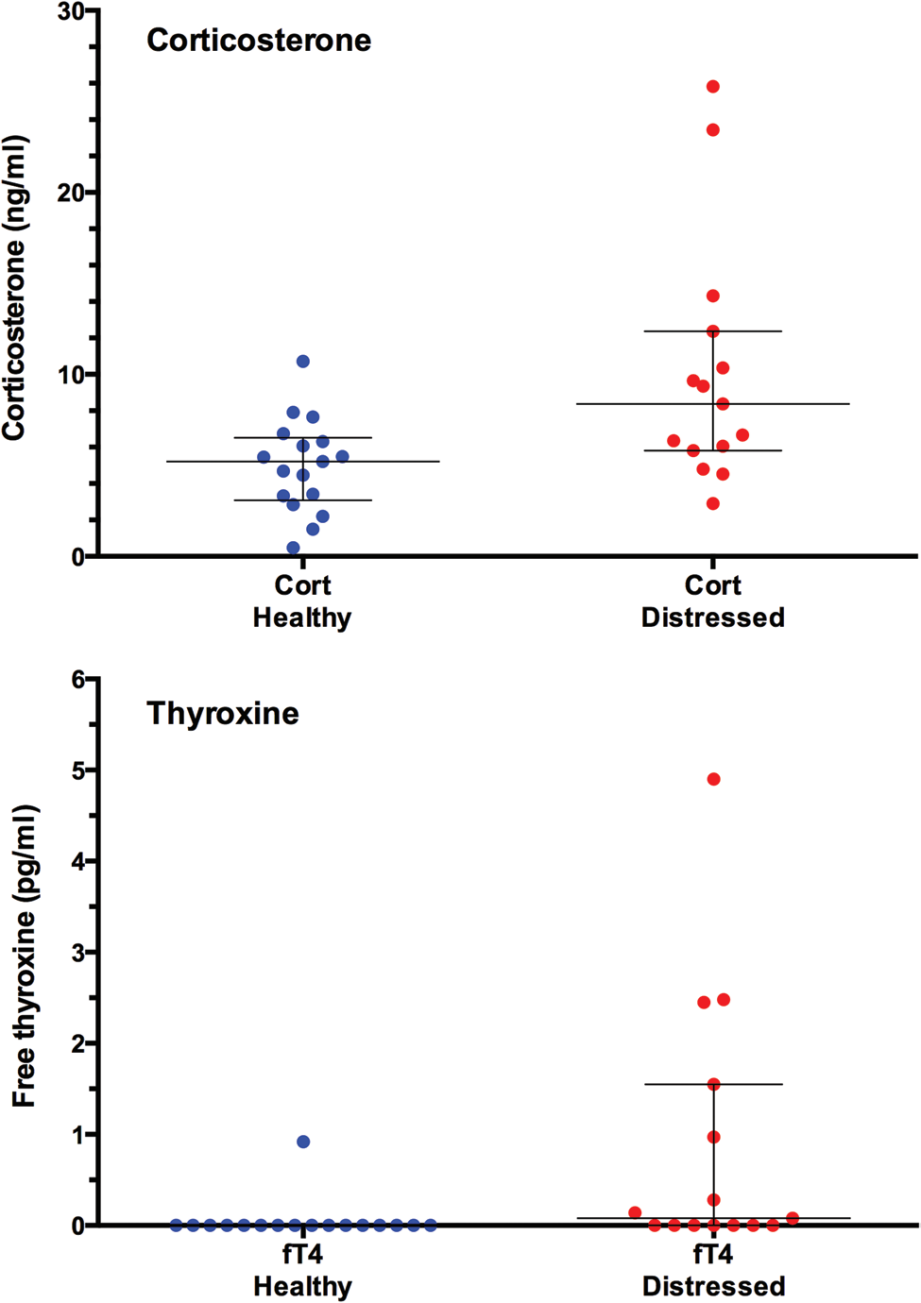
Corticosterone (Cort; top panel) and free thyroxine (fT4; bottom panel) in 17 healthy leatherback turtles (‘Release’ samples only) and 15 distressed leatherback turtles. Scatterplots show all data points; horizontal bars are medians, and whiskers are interquartiles. Corticosterone and fT4 concentrations were both significantly higher in distressed turtles than in healthy turtles.

Corticosterone and fT4 concentrations were both significantly higher in distressed turtles than in healthy turtles (corticosterone: distressed turtles, mean = 10.05 ± 1.72 ng/ml, median = 8.38 ng/ml; and healthy turtles, mean = 4.97 ± 0.62 ng/ml, median = 5.21 ng/ml; *t*_30_ = 3.133, *P* = 0.0039; and fT4: distressed turtles, mean = 0.86 ± 0.37 pg/ml, median = 0.08 pg/ml; and healthy turtles, mean = 0.05 ± 0.05 pg/ml, median = 0.00 pg/ml; *U* = 66, *P* = 0.0028; Fig. 1).

**Figure 2:**
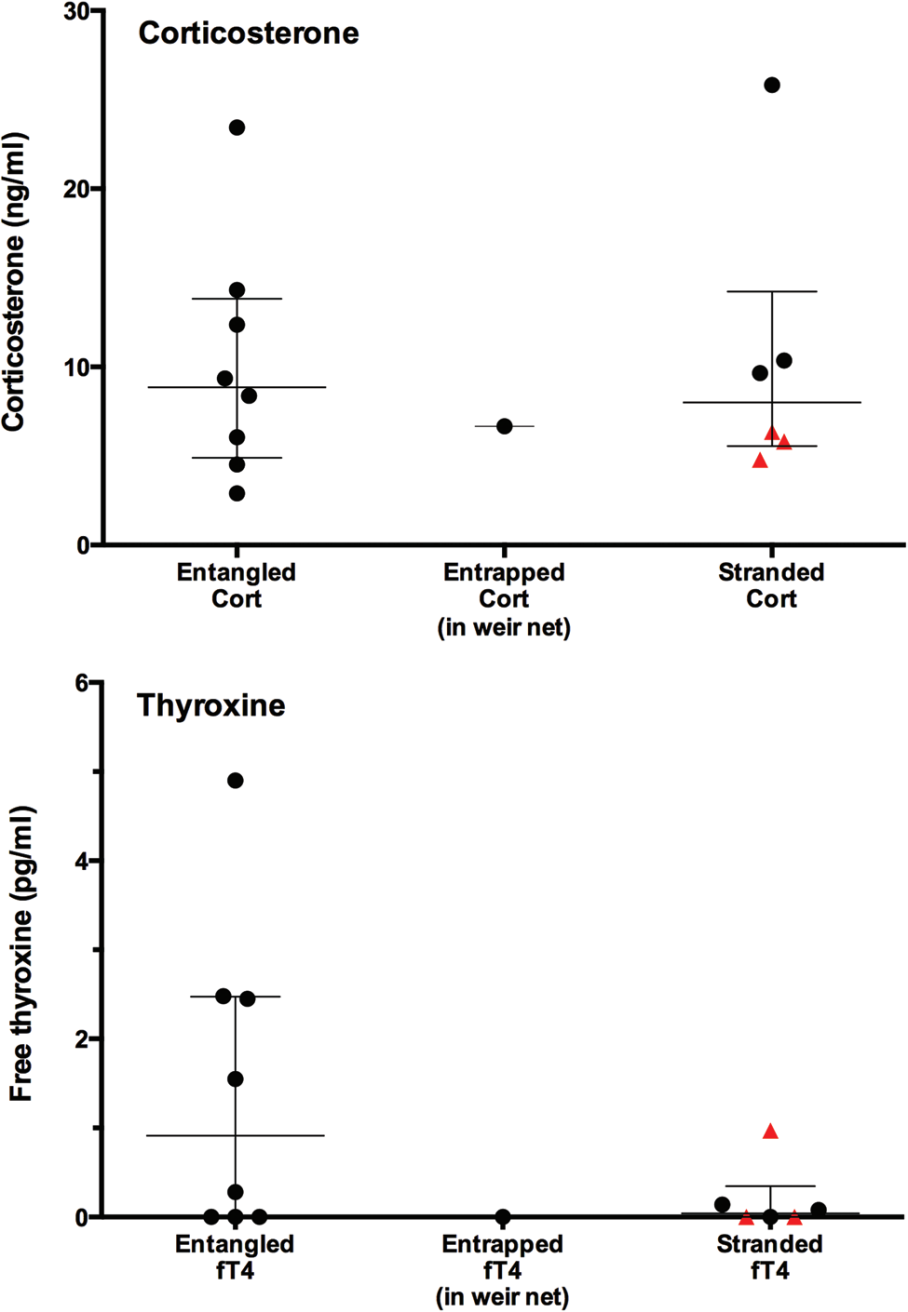
Corticosterone (Cort; top panel) and free thyroxine (fT4; bottom panel) in leatherback turtles entangled in fishing gear (*n* = 8), entrapped in a weir net (*n* = 1) or stranded on shore (*n* = 6). Red triangles indicate moribund turtles. Scatterplots show all data points; horizontal bars are medians, and whiskers are interquartiles. There were no significant differences in either hormone among the three groups.

Within the distressed group, hormone concentrations were similar regardless of the nature of the stressor, i.e. entanglement, entrapment or stranding (entangled turtles, corticosterone, mean = 10.16 ± 2.33 ng/ml, median = 8.87 ng/ml; fT4, mean = 1.46 ± 0.62 pg/ml, median = 0.92 pg/ml; single entrapped turtle, corticosterone = 6.67 ng/ml, fT4 non-detectable; and stranded turtles, corticosterone, mean = 10.46 ± 3.2 ng/ml, median = 8.00 ng/ml; fT4, mean = 0.20 ± 0.16 pg/ml, median = 0.04 pg/ml; Fig. 2). There were no significant statistical differences in entangled vs. stranded turtles for either hormone (corticosterone, *t*_12_ = 0.08926, *P* = 0.9303; and fT4, *U* = 14.50, *P* = 0.2188). However, within the stranded group (*n* = 6), there appeared to be a relationship between corticosterone and clinical state; the three stranded turtles that were responsive when found all had relatively elevated corticosterone, whereas the three moribund turtles all had lower corticosterone (Fig. 2). Owing to low sample sizes, a statistical comparison between these two subgroups could not be performed.

### Relationship of corticosterone with handling time

**Figure 3:**
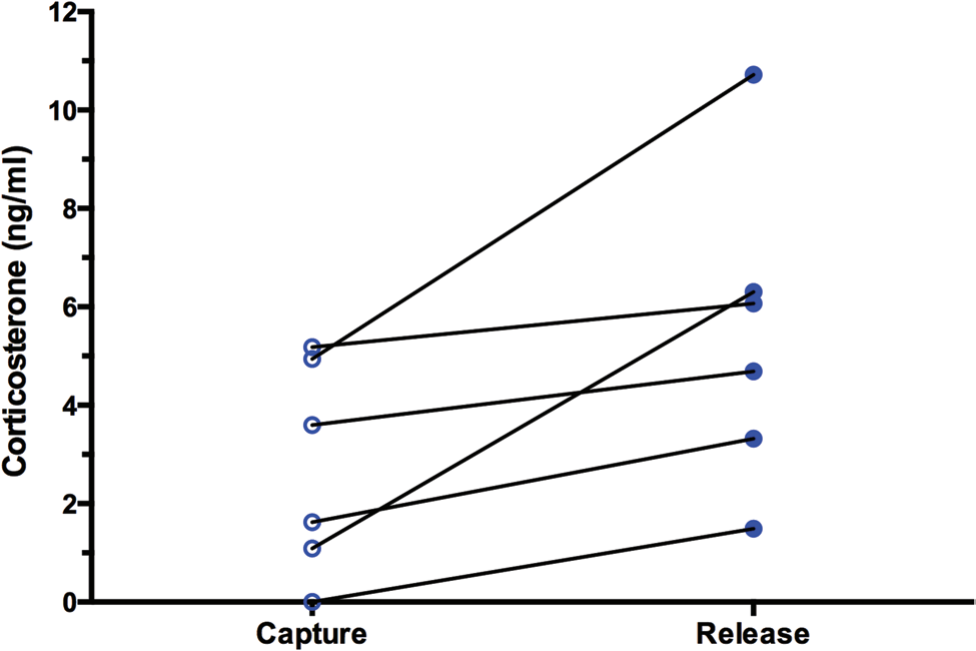
Significant increase in corticosterone over time in six leatherback turtles sampled immediately after capture (left) and ∼25 min later, immediately before release (right). Lines connect samples from the same turtle.

**Figure 4:**
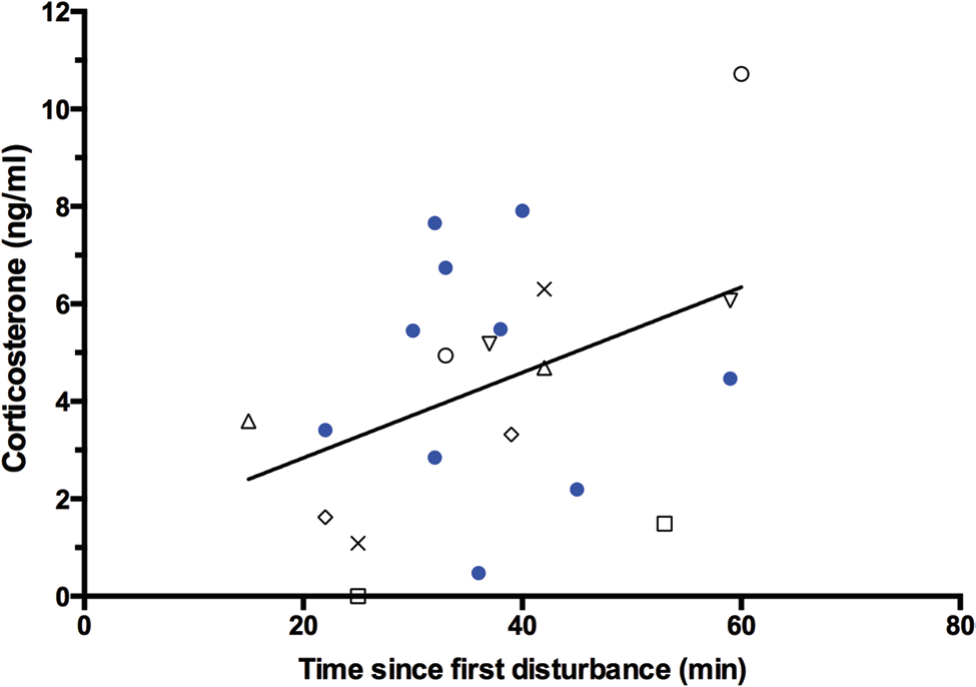
Significant positive relationship of corticosterone with time since first disturbance in 17 healthy leatherback turtles sampled at various times after capture. Six turtles contributed two samples to this data set; these turtles are each depicted with unique uncoloured symbols. Other turtles (shown as filled coloured circles) were all sampled once. The best-fit linear regression line (all samples included) is shown.

In the six healthy leatherbacks that were sampled twice, corticosterone was always higher in the Release sample compared with that turtle’s Capture sample, and this difference was significant (Capture, 2.74 ± 0.88 ng/ml; Release, 5.43 ± 1.29 ng/ml; *t*_5_ = 3.545, *P* = 0.0083; Fig. 3). Overall, combining all Capture and Release samples from all turtles, there was a significant positive relationship between corticosterone and handling time (*F*_1,20_ = 3.965, *P* = 0.0302, *r*^2^ = 0.1655, *y*-intercept = 1.1 ng/ml; Fig. 4, all symbols). This relationship was primarily driven by the turtles sampled twice; when the analysis was restricted to only the turtles sampled twice, the relationship between corticosterone and handling time became stronger (*F*_1,11_=6.065, *P* = 0.0158, *r*^2^ = 0.3554; Fig. 4, open symbols). Conversely, in the turtles sampled only once (at the end of handling), there was no significant relationship of corticosterone with handling time (*F*_1,7_ = 0.0111, *P* = 0.4595, *r*^2^ = 0.0016; Fig. 4, filled blue circles).

## Discussion

Plasma corticosterone concentrations were significantly higher in distressed leatherbacks than in healthy leatherbacks, even though the healthy leatherbacks were sampled at the end of a relatively prolonged (up to 1 h) capture and handling event. Generally, healthy leatherbacks at the end of handling had circulating corticosterone of ∼5 ng/ml, with some individuals reaching the 8–10 ng/ml range. In contrast, distressed leatherbacks had average corticosterone ∼2-fold higher (∼10 ng/ml), and in some individuals the corticosterone concentrations exceeded 20 ng/ml. It is possible that these results do not reflect maximal endocrine responses in light of the unknown duration of entanglement of stranding for these individuals.

In a limited sample size of turtles sampled twice (*n* = 6 turtles), the Release sample always had higher corticosterone than the Capture sample. Corticosterone concentrations in the second sample were generally in the range of 5–10 ng/ml, and the turtle with the highest corticosterone also experienced the longest handling event (1 h). These data confirm that leatherbacks were mounting an adrenal response to handling. Generally, corticosterone concentrations in these second samples approached the lower range of that seen in alert stranded leatherbacks. Clinical data on these same turtles, however (presented in a separate study; Innis *et al.*, 2014), indicate that the healthy turtles were still within normal bounds physiologically. For example, there was no significant elevation in glucose (Innis *et al.*, 2014), an analyte that tends to increase during prolonged capture and handling events in other sea turtle species (Aguirre *et al.*, 1995; Hoopes *et al.*, 2000; Gregory and Schmid, 2001; Hunt *et al.*, 2016).

The significant linear relationship observed between corticosterone and handling time suggests that, at least in the turtles sampled twice, corticosterone continued to increase throughout the time period studied and might not have peaked before the handling event ended. In other sea turtle species studied during longer capture events, peak corticosterone is typically not attained until 4–8 h after capture (green turtle, *Chelonia mydas*, Aguirre *et al.*, 1995; Jessop and Hamann, 2005; hawksbill turtle, *Eretmochelys imbricata*, Jessop *et al.*, 2004c; loggerhead turtle, *Caretta caretta*, Morris, 1982; Blanvillain *et al.*, 2008; olive ridley, *Lepidochelys olivacea*, Valverde *et al.*, 1999). Likewise, in studies of sea turtles using capture stress protocols that lasted an hour or less, the maximal observed corticosterone has typically been measured in the last sample (e.g. Gregory and Schmid, 2001; Snoddy *et al.*, 2009), as was the case here. Given these patterns, we suspect that it is likely that corticosterone in leatherback turtles may continue to increase for hours after capture. It is also possible that corticosterone could reach an asymptote, as suggested by the lack of relationship in the subgroup of turtles that was sampled only once at the end of handling; this subgroup of turtles might already have attained peak corticosterone. However, sample size was small for this analysis and statistical power may have been limited. Generally, based on the patterns seen in turtles sampled twice, and in light of previously reported increases in blood potassium concentration in leatherbacks during some capture events (Harris *et al.*, 2011, Innis *et al.*, 2014), we continue to recommend close physiological monitoring during leatherback capture events, including assessment of cardiac and respiratory rate, body temperature and blood pH, gases and electrolytes. We suggest that long-duration capture events (>1 h) be undertaken with caution.

### Estimating baseline corticosterone in leatherbacks

Baseline corticosterone has not been determined for leatherback turtles. Only two published studies report plasma corticosterone concentrations of this species, and both studies focused on nesting females sampled on land. Deem *et al.* (2006) found that nesting leatherback females sampled during the egg-laying trance almost always had undetectable corticosterone, but given that the egg-laying trance in sea turtles is thought to involve adrenal suppression (e.g. Jessop *et al.*, 1999b; Valverde *et al.* 1999) this result may not be generalizable to other age/sex classes. Rostal *et al.* (2001) reported that nesting female leatherbacks sampled upon emergence from the water (but not yet in the trance state) had plasma corticosterone of 1.4–3.9 ng/ml; however, in that study the capture method, sampling protocol and time since first disturbance were not described, so it is unclear whether capture and handling might have affected results (Rostal *et al.*, 2001). A rough estimate of baseline corticosterone can be derived from the data presented here via extrapolation of the linear regression line shown in Fig. 4 to the *y*-axis intercept, i.e. a hypothetical corticosterone concentration of 1.1 ng/ml at time zero. This approach relies on two assumptions: first, that the turtles were not yet at peak corticosterone (which, given the lack of relationship of corticosterone with handling time for turtles sampled once, might not be the case); and second, that the early phase of corticosterone elevation is linear or close to linear. The kinetics of the first hour of the corticosterone increase have not been well delineated for sea turtles, largely because the majority of sea turtle stress studies have only sampled the animals a few minutes after capture and again at 1 h or more post-capture, but not at any time points in between (e.g. Aguirre *et al.* 1995; Gregory *et al.*, 1996; Ortiz *et al.*, 2000; Jessop *et al.*, 2004b, c; Jessop and Hamann, 2005; Snoddy *et al.*, 2009). Two studies that did include additional sampling time points are those of Gregory and Schmid (2001), which included a 30 min time point for Kemp’s ridley turtles, and Valverde *et al.* (1999), which included time points at 20 and 40 min for olive ridley turtles. Both these studies indicated a roughly linear or close to linear elevation of corticosterone during the first hour, but data analysis did not concentrate on the slope of the increase. Generally, however, an estimated baseline corticosterone of ∼1 ng/ml for leatherbacks is in rough agreement with baselines of 1–2 ng/ml commonly reported from other sea turtle species (flatback turtle, *Natator depressus*, Ikonomopoulou *et al.*, 2014; green turtle, Jessop *et al.*, 1999a, b, 2000, 2004a, b; Jessop and Hamann, 2004; Rostal *et al.*, 2001; hawksbill turtle, Jessop *et al.*, 2004c; loggerhead turtle, Gregory *et al.*, 1996; Whitter *et al.*, 1997; Blanvillain *et al.*, 2008; Valente *et al.*, 2009; olive ridley turtle, Valverde, 1996; Valverde *et al.*, 1999). We emphasize, however, that our estimate of ∼1 ng/ml for potential baseline corticosterone in leatherbacks is intended only as a rough estimate and is presented here primarily in order to spur further research. More data from leatherback turtles will be necessary to determine baseline corticosterone in this species.

### Interpretation of elevated corticosterone

Stress-induced elevations in corticosterone can be difficult to evaluate in terms of potential impact on health or potential long-term detriments (e.g. to growth or reproduction). In other sea turtle species, substantial variation in adrenal activation and circulating corticosterone can occur with season, sex, age class and reproductive state (see citations above). Furthermore, details of handling protocols, extraction steps and assay methodology can add substantial additional variation. Despite these differences in methodology, some patterns are apparent from a review of the literature, and it may be possible that rough assessments can be made as to whether a given stressor indicates mild or severe stress, at least in relationship to the degree of observed adrenal activation. Generally, stress-induced elevations in corticosterone that remain <10 ng/ml, or roughly five times baseline, have typically been reported to occur during relatively mild events (e.g. turtle out of water but kept cool and not handled; turtle placed in novel tank; turtle transported for short periods; Aguirre *et al.*, 1995; Gregory and Schmid, 2001; Hunt *et al.*, 2016). Corticosterone concentrations >10 ng/ml, or ∼10-fold above baseline, have often been reported from turtles exposed to moderate stressors, such as capture in 15 min trawls, heat stress, laparoscopy or prolonged (>24 h) transport in a vehicle (Valverde, 1996; Valverde *et al.*, 1999; Jessop *et al*., 2000, 2004b; Blanvillain *et al.*, 2008; Hunt *et al.*, 2016). Corticosterone concentrations >20 ng/ml have been reported in only a few studies, most often in response to life-threatening stressors or combinations of multiple stressors, such as prolonged time in tangle nets (Gregory and Schmid, 2001), cold-stunning combined with stranding and transport (Hunt *et al.*, 2012) or trawl-net capture combined with heat stress (Gregory *et al.*, 1996). In some of these latter cases, individual turtles have been reported with corticosterone >50 ng/ml. Although comparisons across species and even across individuals are difficult to interpret, the corticosterone concentrations observed here in healthy leatherbacks appear in line with ‘mild’ stress, whereas the distressed leatherbacks spanned a range of corticosterone concentrations indicative of moderate to severe stress.

### Free thyroxine

Free thyroxine was undetectable in almost all healthy leatherbacks, but was detectable in the majority of entangled leatherbacks and in several stranded leatherbacks. The higher concentrations of fT4 seen in distressed leatherbacks were somewhat surprising, because stress is generally thought to inhibit thyroid hormone secretion (Wilber and Utiger, 1969; Eales, 1988; St Aubin and Geraci, 1992; Mastorakos and Pavlatou, 2005; Norris, 2006; Nadolnik, 2011). In cold-stunned Kemp’s ridley turtles, for example, fT4 and corticosterone are inversely correlated, with fT4 strongly suppressed in stressed animals that are newly cold-stunned and elevating several-fold as turtles recover (Hunt *et al.*, 2012). However, some stressors that entail increases in energetic output (e.g. ‘exercise stress’) have been reported to have different relationships with thyroid hormones (Uribe *et al.*, 2014). Entanglement and stranding may be energetically expensive stressors. Entangled whales, for example, are thought to expend considerable extra effort to swim while dragging fishing gear (van der Hoop *et al.*, 2013). Entangled leatherback turtles do show some indications of altered metabolic status and high energetic burden, such as significantly elevated plasma β-hydroxybutyrate concentrations and decreased plasma urea and triglyceride concentrations (Innis *et al.*, 2010). Stranded turtles, too, may have to expend extra energy for respiration and locomotion out of the water; the energetic burden of stranding might be particularly great for leatherbacks because of their very large body mass. The only other published data on thyroxine in leatherbacks are from one study on nesting females (which, like stranded animals, must ambulate and respire out of the water). Females in that study had total T4 (free + bound) concentrations averaging 6.0 ng/ml (Perrault *et al.*, 2012). However, fT4 was not quantified (J.R. Perrault, personal communication), and given that total T4 data are not directly comparable with fT4 data (i.e. the present study) it is unclear whether nesting females have elevated thyroid hormone concentrations. Generally, however, our data support an interpretation that some types of stressors may involve increases in thyroid hormones and/or in metabolic rate.

It is possible that the varied body temperatures of turtles in the present study might have influenced hormone concentrations. Moon (1992) and Hunt *et al.* (2012) demonstrated decreased thyroid hormone concentrations (but increased corticosterone) in hard-shelled sea turtle species exposed to low environmental temperatures. Such studies have not been conducted for leatherback turtles to date. The majority of turtles in the present study had very similar body temperatures in the range of 25–30°C, whereas only four turtles, all of which were stranded, had a body temperature <23°C. Thus, for the stranded turtles, it is difficult to separate the potential effect of temperature from other stranding-related factors. In many reptiles, corticosterone and thyroid hormone concentrations have been reported to change with temperature, or at least to correlate with seasonal changes in ambient temperature (e.g. Licht *et al.*, 1989; Dupoué *et al.*, 2013; Anderson *et al.*, 2015). Such temperature-related responses can be species specific and have been suggested to be adaptive responses to the thermal environment (e.g. Telemeco and Addis, 2014), but it is also possible that these endocrine changes might simply be attributable to non-adaptive temperature effects on secretion rate and clearance rate. The influence of body temperature on the endocrine status of leatherback turtles is worthy of further study.

Cumulatively, previous data (Innis *et al.*, 2010) and the data presented here suggest that distressed leatherback turtles use a variety of physiological mechanisms, including adrenal and thyroid responses, to mobilize energy during stressful events. It is possible that exhaustion of these adaptive mechanisms, combined with other physiological and physical sequelae (e.g. hyperkalaemia, mechanical injury and inhalation of sea water), contributes to the eventual death of distressed leatherback turtles (Innis *et al*., 2014).

### Conclusions

In conclusion, the work here represents the first study of stress-related hormones in non-nesting adult leatherbacks. Live capture and handling of leatherbacks at sea (using the methods outlined here, and with total event duration generally <1 h), appears to elicit a fairly mild adrenal response, whereas entanglement and stranding result in more pronounced adrenal activation. Further study of thyroid hormones and their possible role in managing energetically demanding stressors would be informative. Basic descriptive data on baseline corticosterone, peak corticosterone and the temporal characteristics of the adrenal stress response would also be useful. Timed serial blood sampling (e.g. more than two samples and, ideally, covering a longer time span) of adult live leatherbacks during capture events would be especially useful for investigation of the endocrine and other physiological responses of this species to capture and to other anthropogenic stressors. Future endocrine studies in this species should consider a fully prospective study design, but if prospective study is not feasible, we suggest that researchers should collect and archive plasma samples whenever possible, and we encourage field researchers to note the time of first disturbance and sample collection times. Such studies will improve our understanding of the stress physiology of leatherback turtles, enabling further optimization of handling protocols and clinical care decisions, as well as management decisions related to research capture methods, fisheries interactions and other anthropogenic stressors.

## Funding

This work was supported by the National Oceanographic and Atmospheric Administration (grants #NA04NMF4550391, #NA10NMF4720028 and #EM113F08SE3672), the National Fish and Wildlife Foundation (grants #2008-0076-000 and #2003-0206-014), the Cape Cod Commercial Hook Fishermen’s Association and the Marine Conservation Action Fund.

## References

[COW022C1] Aguirre AA, Balazs GH, Spraker TR, Gross TS (1995) Adrenal and hematological responses to stress in juvenile green turtles (*Chelonia mydas*) with and without fibropapillomas. Physiol Zool 68: 831–854.

[COW022C2] Alfaro-Shigueto J, Dutton P, Van Bressem M, Mangel J (2007) Interactions between leatherback turtles and Peruvian artisanal fisheries. Chelonian Conserv Biol 6: 129–134.

[COW022C3] Anderson L, Nelson N, Cree A (2015) Glucocorticoids in tuatara (*Sphenodon punctatus*): some influential factors, and applications in conservation management. Gen Comp Endocrinol in press, doi:10.1016/j.ygcen.2015.12.001. 10.1016/j.ygcen.2015.12.001 26673869

[COW022C4] Ayres KL, Booth RK, Hempelmann JA, Koski KL, Emmons CK, Baird RW, Balcomb-Bartok K, Hanson MB, Ford MJ, Wasser SK (2012) Distinguishing the impacts of inadequate prey and vessel traffic on an endangered killer whale (*Orcinus orca*) population. PLoS ONE 7: e36842. 22701560 10.1371/journal.pone.0036842 PMC3368900

[COW022C5] Benson S, Eguchi T, Foley D, Forney K, Bailey H, Hitipeuw C, Samber B, Tapilatu R, Rei V, Ramohia P (2011) Large-scale movements and high-use areas of western Pacific leatherback turtles, *Dermochelys coriacea*. Ecosphere 2: art84.

[COW022C6] Bentley PJ (1998) Comparative Vertebrate Endocrinology. Cambridge University Press, Cambridge, UK.

[COW022C7] Blanvillain G, Pease AP, Segars AL, Rostal DC, Richards AJ, Owens D (2008) Comparing methods for the assessment of reproductive activity in adult male loggerhead sea turtles *Caretta caretta* at Cape Canaveral, Florida. Endang Species Res 6: 75–85.

[COW022C8] Dantzer B, Fletcher Q, Boonstra R, Sheriff M (2014) Measures of physiological stress: a transparent or opaque window into the status, management and conservation of species? Conserv Physiol 2(1): cou023; doi:10.1093/conphys/cou023. 10.1093/conphys/cou023 PMC4732472 27293644

[COW022C9] Deem SL, Dierenfeld ES, Sounguet GP, Alleman AR, Cray C, Poppenga RH, Norton TM, Karesh WB (2006) Blood values in free-ranging nesting leatherback sea turtles (*Dermochelys coriacea*) on the coast of the republic of Gabon. J Zoo Wildl Med 37: 464–471. 17315430 10.1638/05-102.1

[COW022C10] Dodge K, Logan J, Lutcavage M (2011) Foraging ecology of leatherback sea turtles in the Western North Atlantic determined through multi-tissue stable isotope analyses. Mar Biol 158: 2813–2824.

[COW022C11] Dodge K, Galuardi B, Miller T, Lutcavage M (2014) Leatherback turtle movements, dive behavior, and habitat characteristics in ecoregions of the Northwest Atlantic Ocaen. PLoS ONE 9: e91726. 24646920 10.1371/journal.pone.0091726 PMC3960146

[COW022C12] Dodge KD, Galuardi B, Lutcavage M (2015) Orientation behaviour of leatherback sea turtles within the North Atlantic subtropical gyre. Proc Biol Sci 282 (Suppl): 20143129. 25761714 10.1098/rspb.2014.3129 PMC4375880

[COW022C130] Dupoué A, Brischoux F, Lourdais O, Angelier F (2013) Influence of temperature on the corticosterone stress-response: An experiment in the Children’s python (Antaresia childreni). Gen Comp Endocrinol 193: 178–184. 23948369 10.1016/j.ygcen.2013.08.004

[COW022C13] Doyle T, Houghton J, Davenport J, Hays G (2008) Leatherback turtles satellite tagged in European waters. Endang Species Res 4: 23–31.

[COW022C14] Eales JG (1988) The influence of nutritional state on thyroid function in various vertebrates. Am Zool 28: 351–362.

[COW022C15] Ezan E, Grassi J (2000) Optimization. In Gosling JP, ed., Immunoassays: a Practical Approach. Oxford University Press, Oxford, UK, pp 187–210.

[COW022C16] Frair W, Ackman RG, Mrosovsky N (1972) Body temperature of *Dermochelys coriacea*: warm turtle from cold water. Science 177: 791–793. 17840128 10.1126/science.177.4051.791

[COW022C17] Gerwien RW, John-Alder HB (1992) Growth and behavior of thyroid-deficient lizards (*Sceloporus undulatus*). Gen Comp Endocrinol 87: 312–324. 1398024 10.1016/0016-6480(92)90036-j

[COW022C18] Godley B, Gaywood M, Law R, McCarthy C, McKenzie C, Patterson I, Penrose R, Reid R, Ross H (1998) Patterns of marine turtle mortality in British waters (1992–1996) with reference to tissue contaminant levels. J Mar Biol Assoc (UK) 78: 973–984.

[COW022C19] Graham KM, Mylniczenko ND, Burns CM, Bettinger TL, Wheaton CJ (2016) Examining factors that may influence accurate measurement of testosterone in sea turtles. J Vet Diagn Invest 28: 12–19. 26699527 10.1177/1040638715618989

[COW022C20] Gregory LF, Schmid JR (2001) Stress responses and sexing of wild Kemp’s ridley sea turtles (*Lepidochelys kempii*) in the northeastern Gulf of Mexico. Gen Comp Endocrinol 124: 66–74. 11703072 10.1006/gcen.2001.7683

[COW022C21] Gregory LF, Gross TS, Bolten AB, Bjorndal KA, Guillette LJ Jr (1996) Plasma corticosterone concentrations associated with acute captivity stress in wild loggerhead sea turtles (*Caretta caretta*). Gen Comp Endocrinol 104: 312–320. 8954764 10.1006/gcen.1996.0176

[COW022C22] Grotjan HE, Keel BA (1996) Data interpretation and quality control. In Diamandis EP, Christopoulos TK, eds, Immunoassay. Academic Press, San Diego, pp 51–95.

[COW022C23] Harris H, Benson S, Gilardi K, Poppenga R, Dutton P, Work T, Mazet J (2011) Comparative health assessment of western Pacific leatherback turtles (*Dermochelys coriacea*) foraging off the coast of California: 2005–2007. J Wildl Dis 47: 321–337. 21441185 10.7589/0090-3558-47.2.321

[COW022C24] Hoopes LA, Landry AMJ, Stabenau EK (2000) Physiological effects of capturing Kemp’s ridley sea turtles, *Lepidochelys kempii*, in entanglement nets. Can J Zool 78: 1941–1947.

[COW022C25] Hulbert AJ (2000) Thyroid hormones and their effects: a new perspective. Biol Rev 75: 519–631. 11117200 10.1017/s146479310000556x

[COW022C26] Hunt KE, Trites A, Wasser SK (2004) Validation of fecal glucocorticoid analyses for Steller sea lions (*Eumetopias jubatus*) by adrenocorticotropic hormone (ACTH) challenge. Physiol Behav 80: 595–601. 14984791 10.1016/j.physbeh.2003.10.017

[COW022C27] Hunt K, Innis CJ, Rolland RM (2012) Corticosterone and thyroxine in cold-stunned Kemp’s ridley sea turtles (*Lepidochelys kempii*). J Zoo Wildl Med 43: 479–493. 23082511 10.1638/2011-0149R1.1

[COW022C28] Hunt KE, Innis CJ, Kennedy AE, McNally KL, Davis DG, Burgess EA, Merigo C (2016) Assessment of ground transportation stress in juvenile Kemp’s ridley sea turtles (*Lepidochelys kempii*). Conserv Physiol 4(1): cov071; doi:10.1093/conphys/cov071. 10.1093/conphys/cov071 PMC4804726 27293750

[COW022C29] Ikonomopoulou MP, Bradley AJ, Ibrahim K, Limpus CJ, Fernandez-Rojo MA, Vagenas D, Whittier JM (2014) Hormone and metabolite profiles in nesting green and flatback turtles: turtle species with different life histories. Adv Zool 2014: 503209 doi:10.1155/2014/503209.

[COW022C30] Innis CJ, Merigo C, Dodge KD, Tlusty M, Dodge M, Sharp B, Myers A, McIntosh A, Wunn DS, Perskins C et al (2010) Health evaluation of leatherback turtles (*Dermochelys coriacea*) in the northwestern Atlantic during direct capture and fisheries gear disentanglement. Chelonian Conserv Biol 9: 205–222.

[COW022C31] Innis CJ, Cavin JM, Merigo C, Sampson KA, Dodge KL, Dodge MF (2013) Medical management and post-release monitoring of a stranded leatherback turtle (*Dermochelys coriacea*). IAAAM 44th Annual Conference Proceedings, Sausalito, CA, pp 77–78.

[COW022C32] Innis CJ, Merigo C, Cavin JM, Hunt KE, Dodge KL, Lutcavage M (2014) Serial assessment of the physiological status of leatherback turtles (*Dermochelys coriacea*) during direct capture events in the northwestern Atlantic Ocean: comparison of post-capture and pre-release data. Conserv Physiol 2(1): cou048; doi:10.1093/conphys/cou048. 10.1093/conphys/cou048 PMC4806728 27293669

[COW022C33] James M, Mrosovsky N (2004) Body temperatures of leatherback turtles (*Dermochelys coriacea*) in temperate waters off Nova Scotia, Canada. Can J Zool 82: 1302–1306.

[COW022C34] James M, Ottensmeyer C, Myers R (2005) Identification of high-use habitat and threats to leatherback sea turtles in northern waters: new directions for conservation. Ecol Lett 8: 195–201.

[COW022C35] James M, Sherrill-Mix S, Myers R (2007) Population characteristics and seasonal migrations of leatherback sea turtles at high latitudes. Mar Ecol Prog Ser 337: 245–254.

[COW022C36] Jessop TS, Hamann M (2004) Hormonal and metabolic responses to nesting activities in the green turtle, *Chelonia mydas*. J Exp Mar Biol Ecol 308: 253–267.

[COW022C37] Jessop TS, Hamann M (2005) Interplay between age class, sex and stress response in green turtles (*Chelonia mydas*). Aust J Zool 53: 131–136.

[COW022C38] Jessop TS, FitzSimmons NN, Limpus CJ, Whittier JM (1999a) Interactions between behavior and plasma steroids within the scramble mating systems of the promiscuous green turtle, *Chelonia mydas*. Horm Behav 36: 86–97. 10506533 10.1006/hbeh.1999.1527

[COW022C39] Jessop TS, Limpus CJ, Whittier JM (1999b) Plasma steroid interactions during high-density green turtle nesting and associated disturbance. Gen Comp Endocrinol 115: 90–100. 10375467 10.1006/gcen.1999.7288

[COW022C40] Jessop TS, Hamann M, Read MA, Limpus CJ (2000) Evidence for a hormonal tactic maximizing green turtle reproduction in response to a pervasive ecological stressor. Gen Comp Endocrinol 118: 407–417. 10843792 10.1006/gcen.2000.7473

[COW022C41] Jessop TS, Hamann M, Limpus CJ (2004a) Body condition and physiological changes in male green turtles during breeding. Mar Ecol Prog Ser 276: 281–288.

[COW022C42] Jessop TS, Sumner JM, Lance V, Limpus CJ (2004b) Reproduction in shark-attacked sea turtles is supported by stress-reduction mechanisms. Proc Biol Sci 271 (Suppl): S91–S94. 15101429 10.1098/rsbl.2003.0102 PMC1809973

[COW022C43] Jessop TS, Sumner JM, Limpus CJ, Whittier JM (2004c) Interplay between plasma hormone profiles, sex and body condition in immature hawksbill turtles (*Eretmochelys imbricata*) subjected to a capture stress protocol. Comp Biochem Physiol A Mol Integr Physiol 137: 197–204. 14720605 10.1016/j.cbpb.2003.09.029

[COW022C44] John-Alder HB (1990) Thyroid regulation of resting metabolic rate and intermediary metabolic enzymes in a lizard (*Sceloporus occidentalis*). Gen Comp Endocrinol 77: 52–62. 2295423 10.1016/0016-6480(90)90205-z

[COW022C45] Joly K, Wasser SK, Booth R (2015) Non-invasive assessment of the interrelationships of diet, pregnancy rate, group composition, and physiological and nutritional stress of barren-ground caribou in late winter. PLoS ONE 10: e0127586. 26061003 10.1371/journal.pone.0127586 PMC4464525

[COW022C46] Kohel KA, MacKenzie DS, Rostal DC, Grumbles JS, Lance VA (2001) Seasonality in plasma thyroxine in the desert tortoise, *Gopherus agassizii*. Gen Comp Endocrinol 121: 214–222. 11178887 10.1006/gcen.2000.7595

[COW022C47] Lewison R, Crowder L, Read A, Freeman S (2004) Quantifying the effects of fisheries on threatened species: the impact of pelagic longlines on loggerhead and leatherback sea turtles. Ecol Lett 7: 221–231.

[COW022C48] Licht P, Breitenbach GL, Congdon JD (1985) Seasonal cycles in testicular activity, gonadotropin, and thyroxine in the painted turtle, *Chrysemys picta*, under natural conditions. Gen Comp Endocrinol 59: 130–139. 3926601 10.1016/0016-6480(85)90427-7

[COW022C49] Licht P, Denver RJ, Pavgi S (1989) Temperature dependence of *in vitro* pituitary, testis, and thyroid secretion in a turtle, *Pseudemys scripta*. Gen Comp Endocrinol 76: 274–285. 2512196 10.1016/0016-6480(89)90159-7

[COW022C50] Madliger C, Love O (2015) The need for a predictive, context-dependent approach to the application of stress hormones in conservation. Conserv Biol 28: 283–287. 24283988 10.1111/cobi.12185

[COW022C51] Madliger CL, Cooke SJ, Crespi EJ, Funk JL, Hultine KR, Hunt KE, Rohr JR, Sinclair BJ, Suski CD, Willis CKR et al (2016) Success stories and emerging themes in conservation physiology. Conserv Physiol 4(1): cov057; doi:10.1093/conphys/cov057. 10.1093/conphys/cov057 PMC4922248 27382466

[COW022C52] Mastorakos G, Pavlatou M (2005) Exercise as a stress model and the interplay between the hypothalamus-pituitary-adrenal and the hypothalamus-pituitary-thyroid axes. Horm Metab Res 37: 577–584. 16175498 10.1055/s-2005-870426

[COW022C53] Moon D-Y (1992) The responses of sea turtles to temperature changes; behavior, metabolism and thyroid hormones. PhD dissertation, Texas A&M University, College Station, TX.

[COW022C54] Moon D-Y, Owens DW, MacKenzie DS (1999) The effects of fasting and increased feeding on plasma thyroid hormones, glucose, and total protein in sea turtles. Zool Sci 16: 579–586.

[COW022C55] Morris YA (1982) Steroid dynamics in immature sea turtles. Master’s thesis, Texas A&M University, College Station, TX.

[COW022C56] Nadolnik L (2011) Stress and the thyroid gland. Biochem (Moscow) Suppl Ser B: Biomed Chem 5: 103–112.

[COW022C57] National Oceanographic and Atmospheric Administration (2014) Leatherback turtle (*Dermochelys coriacea*) http://www.nmfs.noaa.gov/pr/species/turtles/leatherback.htm.

[COW022C58] Norris DO (2006) Vertebrate Endocrinology, Ed 4 Academic Press, New York.

[COW022C59] Ortiz RM, Patterson RM, Wade CE, Byers FM (2000) Effects of acute fresh water exposure on water flux rates and osmotic responses in Kemp’s ridley sea turtles (*Lepidochelys kempii*). Comp Biochem Physiol A Mol Integr Physiol 127: 81–87. 10996820 10.1016/s1095-6433(00)00240-3

[COW022C60] Perrault J, Miller D, Eads E, Johnson C, Merrill A, Thompson L, Wyneken J (2012) Maternal health status correlates with nest success of leatherback sea turtles (*Dermochelys coriacea*) from Florida. PLoS ONE 7: e31841. 22359635 10.1371/journal.pone.0031841 PMC3281022

[COW022C61] Rivera S, Lock B (2008) The reptilian thyroid and parathyroid glands. Vet Clin Exotic Anim Pract 11: 163–175. 10.1016/j.cvex.2007.10.002 18165144

[COW022C62] Romero LM, Wingfield JC (2016) Tempests, Poxes, Predators and People: Stress in Wild Animals and How They Cope. Oxford University Press, New York.

[COW022C63] Rostal DC, Grumbles JS, Palmer KS, Lance VA, Spotila JR, Paladino FV (2001) Changes in gonadal and adrenal steroid levels in the leatherback sea turtle (*Dermochelys coriacea*) during the nesting cycle. Gen Comp Endocrinol 122: 139–147. 11316419 10.1006/gcen.2001.7615

[COW022C64] St Aubin D, Geraci J (1992) Thyroid hormone balance in beluga whales, *Delphinapterus leucas*: dynamics after capture and influence of thyrotropin. Can J Vet Res 56: 1–5. 1586888 PMC1263494

[COW022C65] Sapolsky R, Romero LM, Munck AU (2000) How do glucocorticoids influence the stress response? Integrating permissive, suppressive, stimulatory, and preparative actions. Endocr Rev 21: 55–89. 10696570 10.1210/edrv.21.1.0389

[COW022C66] Sheriff M, Dantzer B, Delehanty D, Palme R, Boonstra R (2011) Measuring stress in wildlife: techniques for quantifying glucocorticoids. Oecologia 166: 869–887. 21344254 10.1007/s00442-011-1943-y

[COW022C67] Snoddy JE, Landon M, Blanvillain G, Southwood A (2009) Blood biochemistry of sea turtles captured in gillnets in the lower Cape Fear River, North Carolina, USA. J Wildl Manage 73: 1394–1401.

[COW022C68] Southwood A, Avens L (2010) Physiological, behavioral, and ecological aspects of migration in reptiles. J Comp Physiol B 180: 1–23. 19847440 10.1007/s00360-009-0415-8

[COW022C69] Telemeco RS, Addis EA (2014) Temperature has species-specific effects on corticosterone in alligator lizards. Gen Comp Endocrinol 206: 184–192. 25019656 10.1016/j.ygcen.2014.07.004

[COW022C70] Uribe R, Jaimes-Hoy L, Ramírez-Martínez C, García-Vásquez A, Romero F, Cisneros M, Cote-Vélez A, Charli J-L, Joseph-Bravo P (2014) Voluntary exercise adapts the hypothalamus-pituitary-thyroid axis in male rats. Endocrinology 155: 2020–2030. 24605825 10.1210/en.2013-1724

[COW022C71] Valente ALS, Velarde R, Parga ML, Marco I, Lavin S, Alegre F, Cuenca R (2009) Reproductive status of captive loggerhead sea turtles based on serum levels of gonadal steroid hormones, corticosterone and thyroxin. Vet J 187: 255–259. 20044286 10.1016/j.tvjl.2009.11.022

[COW022C72] Valverde RA (1996) Corticosteroid dynamics in a free-ranging population of olive ridley sea turtles (*Lepidochelys olivacea* Eschscholtz, 1829) at Playa Nancite, Costa Rica, as a function of their reproductive behavior. dissertation, Texas A&M University, College Station, TX.

[COW022C73] Valverde RA, Owens DW, MacKenzie DS, Amoss MS (1999) Basal and stress-induced corticosterone levels in olive ridley sea turtles (*Lepidochelys olivacea*) in relation to their mass nesting behavior. J Exp Zool 284: 652–662. 10531552 10.1002/(sici)1097-010x(19991101)284:6<652::aid-jez7>3.0.co;2-u

[COW022C74] van der Hoop J, Moore M, Fahlman A, Bocconcelli A, George C, Jackson K, Miller C, Morin D, Pitchford T, Rowles T et al (2013) Behavioral impacts of disentanglement of a right whale under sedation and the energetic cost of entanglement. Mar Mamm Sci 30: 282–307.

[COW022C75] Wallace B, Tiwari M, Girondot M (2013). The IUCN Red List of Threatened Species 2013: *Dermochelys coriacea* www.iucnredlist.org.

[COW022C76] Whitter JM, Corrie F, Limpus CJ (1997) Plasma steroid profiles in nesting loggerhead turtles (*Caretta caretta*) in Queensland, Australia: relationship to nesting episode and season. Gen Comp Endocrinol 106: 39–47. 9126464 10.1006/gcen.1996.6848

[COW022C77] Wilber JF, Utiger RD (1969) The effect of glucocorticoids on thyrotropin secretion. J Clin Invest 48: 2096–2103. 4980930 10.1172/JCI106176 PMC297463

[COW022C78] Wingfield JC, Romero LM (2011) Adrenocortical responses to stress and their modulation in free-living vertebrates. Comprehensive Physiology: Handbook of Physiology, The Endocrine System, Coping with the Environment: Neural and Endocrine Mechanisms: Supplement 23: 211–234. doi: 10.1002/cphy.cp070411.

[COW022C79] Wingfield JC, Hunt K, Breuner C, Dunlap K, Fowler GS, Freed L, Lepson J (1997) Environmental stress, field endocrinology, and conservation biology. In Clemmons R, Buchholz R, eds, Behavioral Approaches to Conservation in the Wild. Cambridge University Press, Cambridge, UK, pp 95–131.

